# What do users care about? Research on user behavior of mobile interactive video advertising

**DOI:** 10.1016/j.heliyon.2022.e10910

**Published:** 2022-10-04

**Authors:** Chao Gu, Shuyuan Lin, Jie Sun, Chun Yang, Jiangjie Chen, Qianling Jiang, Wei Miao, Wei Wei

**Affiliations:** a Department of Culture and Arts Management, Honam University, Gwangju, 62399, South Korea; b Department of Media Design, Tatung University, Taipei, 104, Taiwan; c School of Design, Jiangnan University, Wuxi, 214122, China; d School of Textile Garment and Design, Changshu Institute of Technology, Changshu, 215500, China

**Keywords:** Electronic commerce, Interaction design, Media arts, Psychology

## Abstract

Mobile interactive video advertising is a relatively new type of media. A combination of interactivity and narrative is used to tell the video story to the audience. This study studied in three steps on mobile interactive video advertising to determine the design basis that should be paid attention to. Firstly, the research framework is proposed through expert interview and qualitative coding of the interview text. Secondly, research samples are made through surveys and conference discussions. The samples were designed into 30 types containing various interactive narrative and involvement. Finally, the qualitative results are verified in a quantitative way. The results show that in order to improve the audience's purchase intention, more attention should be paid to interactive narrative, subjective video quality assessment, immersion, satisfaction, and product involvement.

## 1 Introduction

Nowadays, although there are various kinds of mobile video ads, the nature of mobile video ads is still linear. When it comes to mobile video ads, interaction is important. Interactive advertising provides opportunities for both two-way and multi-to-multi communication between consumers, brands, and media (Eastin et al., 2011). The active control of the watching process can give the audience more freedom. In the process of video watching, the audience would no longer passively accept a linear timeline, but be able to switch between immersive watching and active control (Afify, 2020). Many researchers believe that interactive experiences are so important that they can even replace traditional linear experiences. Temporal images should be replaced by interactive images, as audience tend to integrate interactivity into video and audio advertisements (Daly, 2010). Under the trend of interactivity, combining traditional broadcast mode with interactive elements has become a popular approach.

In recent years, interactive elements have been attached importance to mobile video advertisements. Media service providers have already noticed the importance of interaction before this. Digital experience and various digital platforms have begun to provide new virtual experience and game experience in more interactive experience models (C. Wang, 2021). After this, researchers defined interactive video as primarily composed of video material and offered special ways to change the process of transmission to allow free exploration (Hassan and Obeidat, 2022). In this process, the interactive video presents a very significant feature, that is, uses video clips as the main means of expression (Afify, 2020). Through the sequential presentation of video clips, mobile interactive video advertisements fully give users the freedom to choose, so that users can participate in the video and make some changes.

As a relatively new media category, in addition to the increasing importance of action and interactivity in recent years, the development of mobile interactive video advertising also needs the cooperation of technological iteration. In fact, the existing commercialized mobile interactive video advertisements has not entered the market for a long time, but it is this short time has shown the potential. Interactive marketing can attract consumers and help to promote the sales of products. Interaction quickly permeates the society and logically changes the world. Behind the explosive commercial power, this new media form has not been widely discussed in the academic level, and a large number of relevant practitioners lack effective theoretical support in their design and production. Parker's study showed that interactive video can provide 32% more memorable experiences than linear video, increase user activity to 591%, and purchase conversion rate is 11 times higher than traditional media (Parker, 2022). Considering the market potential and value of mobile interactive video advertisement, it is urgent and helpful to conduct corresponding research and put forward design suggestions.

Mobile interactive video advertising has an increasingly wide audience and interactive experience favored by users. Audiences gain more flexibility because they can easily watch and even influence the story at their own pace (C.-Y. Su and Chiu, 2021). Because of the development of interactive technology, audiences and manufacturers can communicate in a way that was not possible before. Previous studies have shown that adding interactive elements to the marketing process leads to positive experiences (Hajarian et al., 2021). From the perspective of practical application, mobile interactive video advertising, as a kind of interactive video advertising, has both commercial value and research value. However, this does not mean that interactive technologies are suitable for all kinds of media and application scenarios. For example, the use of interactive technology in romantic relationships or situations can have negative effects (Vaterlaus and Tulane, 2019). Using interactive video in programming education does not improve students’ learning outcomes (Sözeri and Kert, 2021). Therefore, in order to better achieve business purposes, it is necessary to systematically evaluate the application of interactive video technology in the marketing field. This study investigates the audience perception results, proposes the elements that need to be paid attention to in the design process and establishes a theoretical model.

### 1.1 Research purpose

**Figure 1:**
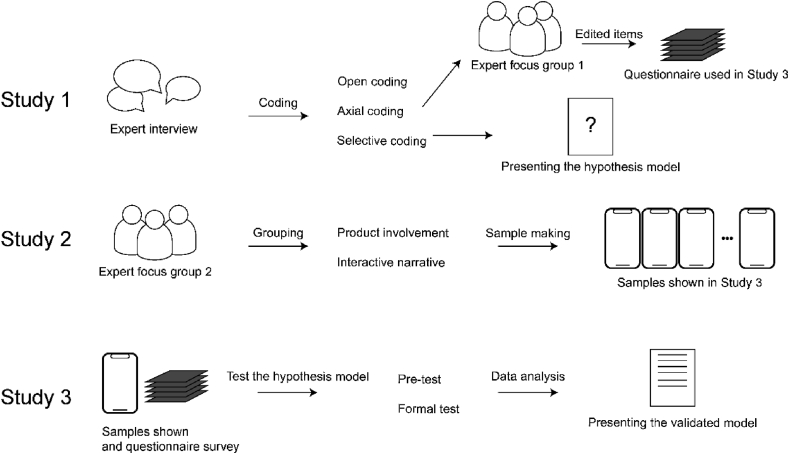
Research procedure.

This study is divided into three sub-studies centering on mobile interactive video advertising, as shown in Figure 1. In sub-study 1, text data were obtained through expert interviews and coded by qualitative methods, and questionnaires and preliminary hypothesis models were established. Sub-study 2 classified product involvement and interactive narrative of products in mobile interactive video advertisements, and made various types of samples according to the classification results. In sub-study 3, participants were invited to watch the samples and answer the questionnaire, and the structural equation model was used to verify the hypothesis model. Through three sub-studies, we propose the mobile interactive mobile advertising awareness model (MIVAPM) in a qualitative and quantitative way.

## 2 Literature review

In this study, ten main dimensions of mobile interactive film advertisement are proposed through interview and qualitative coding, and they are used as control variable or experimental variable model by quantitative method. The dimensions were defined and discussed in previous studies as follows.

### 2.1 Mobile interactive video advertising

**Figure 2:**
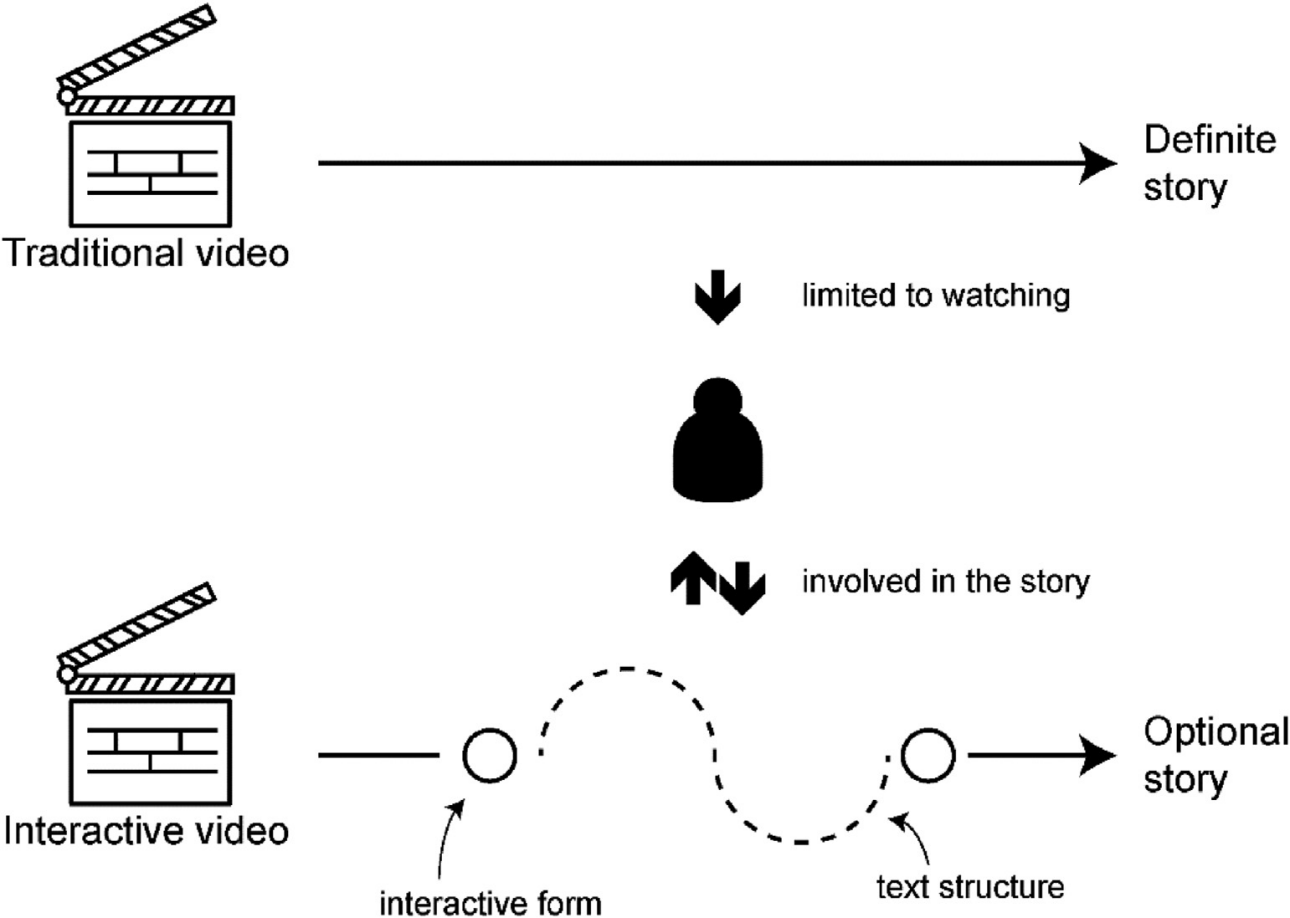
Traditional video versus interactive video.

Interactive video is a form of media that allows viewers to interact with content (Anderson and Davidson, 2019). Different from the traditional linear video, interactive video has multiple interactive nodes in the content. With the development of interactive video technology, audiences are allowed to interact with video in many different ways (Hassan and Obeidat, 2022). Text structure is one of the important attributes of media forms for interactive narration (van der Nat et al., 2021). Therefore, the selection and design of text structure to trigger the story of interactive video, and the sequence of the story can all be affected by the audience. Different works can bring different experiences according to the data exchange of audience interaction and collaboration, which are completely different from traditional videos (Hook, 2018). The comparison between interactive video and traditional video is shown in Figure 2.

Interactivity enables the audience to get access to control the advertising, enhancing the appeal of the advertising to the audience (Pashkevich et al., 2012). Interactive video has great potential in marketing. Interactive video advertising is an effective way to engage consumers because interacting with video can arouse consumers' emotions and their needs (Sedej, 2019). The kind of application provides substantial value for interactive video. It is worth noting that the number and experience of viewers for interactive video viewing using computers and mobile phones may be different. Previous studies have shown that concentration and time distortion play a major role in flow experience when consumers using mobile terminals, while enjoyment is a factor that deserves attention when consumers using PC terminals (Barta et al., 2021). From a device and technology perspective, mobile phones and computers have different hardware types to support the corresponding interactive experience. Gyroscopes, multi-touch screens, and even 5G networks can offer more interaction possibilities for mobile devices (Miao et al., 2021). Nowadays, mobile video marketing is widely regarded as the focus of enterprise strategy design (Mulier et al., 2021). Mobile interactive video advertising is a type of media that has attracted wide attention. Advances in mobile phone and Internet technology have made mobile advertising accessible to viewers in all kinds of surroundings and fragmented hours. Hence, mobile marketing is also getting more and more attention in theoretical research (Hoeck and Spann, 2020).

### 2.2 Content desirability

Content desirability is the extent to which people think the content they are watching is appropriate or desirable (C. C. Su and Chan, 2017). These include images, videos or messages received through social media. Video content plays an important role in the audience's perception, especially in the evaluation of quality (Subramanyam et al., 2020). The audience may overlook the flaws in the picture because of the content. In the process of watching the video, the audience's emotions will be affected by the content, and they will make corresponding facial expressions and physical behaviors (Kortum and Sullivan, 2010). Therefore, in the design of advertising research, we should carefully consider the influence of sample content.

### 2.3 Brand awareness

Brand awareness is a basic and important concept in brand equity (Park and Namkung, 2022). It is usually defined as the ability of consumers to identify and recall brands (Gong et al., 2020). In other words, it can also be defined as the ability to remember brands. brand awareness is often taken into account in consumers' perception and decision-making process. brand awareness is one of the basic conditions affecting consumers' purchasing behavior, and well-known products are more likely to be adopted by consumers (Seo et al., 2020). Popular brand names can help consumers mitigate their perceived risk and form positive evaluations (Ročkutė et al., 2018). Therefore, brand awareness plays a role in giving consumers confidence in the product or service, and the brand appearing in the mobile video advertisement may influence consumers' decision making.

### 2.4 Product involvement

Product involvement is considered to be an evaluation of the importance of a product by consumers considering their own interests, values and needs (Puriwat and Tripopsakul, 2021). This concept has received a lot of attention in the field of consumer behavior. The evaluation of a product involvement is often divided into two categories: highly involved or less involved (Ripoll and Panea, 2019). Consumers with high and low levels of involvement have different reactions to price, provenance, and tag design (Yoon et al., 2019). Products with high involvement are more likely to generate consumer care and enthusiasm, and usually sell more at a higher unit price (Capitello and Sirieix, 2019). Under this premise, consumers may have different feelings to interactive video advertisements with different degrees of involvement.

### 2.5 Interactive narrative

With the development of digitial technology, interactive narrative is rapidly spreading to a variety of media (Koenitz, 2018). This concept is defined as the idea that the development and outcome of a story can be influenced by audience intervention (Cavazza and Young, 2017). In this case, the audience is no longer simply receiving the information conveyed in the media. Participant choice is the main difference between interactive narrative and linear narrative (Robertson et al., 2019). Media with interactive narrative characteristics can carry out real-time two-way input and output with the audience. Particularly in video media, art and technology are intergrated to allow the viewer to enter the story and participate in its development (Andrade et al., 2022). Previous studies have mainly discussed interactivity and narrative planning (Jin et al., 2022). The way the audience participates in the interaction and the arrangement and structure of the story text are two focus parts that constitute the concept of interactive narrative. In particular, educational research has proved that interactive narrative is closely related to the audience's flow experience and will affect their satisfaction (C. Gu et al., 2022). Therefore, for interactive advertising, improving consumers' evaluation of interactive narrative may also be one of the design priorities.

### 2.6 Immersion

Immersion means that the audience is caught up in the story world in a mentally engaged way (Langhof and Güldenberg, 2019). The concept of immersion has received much attention in the field of human-computer interaction. Immersive experience triggers when audience's attention shifts along with the construction of mental representation in the brain (Thon, 2008). At this point the viewer suspends the suspicion that he or she is in a virtual digital environment. This sense of separation from the real world is one of the main characteristics of immersion (Lim et al., 2019). In fact, immersion a state of losing track of time, your surroundings, or yourself (Georgiou et al., 2019). Previous research has shown that immersion can be divided into three levels from shallow to deep (Cheng et al., 2015). The level of total immersion allows the audience to feel immersed in the digital world and empathize with the characters in the story. It is worth noting that there is overlap between immersion state and flow experience in definition, but flow experience is an “all-or-nothing” state (Cairns et al., 2014), while immersion has obvious hierarchical nature. And flow experiences emphasize challenges, while audience immersed in interactive video are not necessarily faced with pressing tasks (Agrewal et al., 2020). From the emotional perspective, the process of achieving immersion is accompanied by recognition and pleasure (Cheng et al., 2015). With this positive feeling, consumers' evaluation of the advertisements and the probability of subsequent purchase behavior may change.

### 2.7 Quality of experience (QoE)

QoE is usually defined as the overall acceptability of the service subjectively perceived by the end user (Mu et al., 2012). According to Kortum and Sullivan (2010), the QoE assessment is divided into two categories: objective video quality assessment (OVQA) and subjective video quality assessment (SVQA). Objective video quality assessment is based on parameter judgment while SVQA is formed by human's real feelings to the image (T.-J. Liu, Lin, Lin and Kuo, 2013). Image size, frame rate, number of colors used to represent the image, encoding format used, dynamic range, constant bit rate and variable bit rate, interlaced versus progressive scan are the parameters that objectively matter (Hooper et al., 2007). In addition, chroma sampling is also one of the parameters affecting the video quality (Seshadrinathan et al., 2010). Relying on objective parameters to improve QoE requires corresponding cost input, and efficiency may decrease with the increase of parameters.

The ultimate audience of the video is people, and the ultimate goal is to gain the audience's perception (X. Gu, Qiu, Feng, Debing and Zhibo, 2012). Therefore, SVQA is important as a judgment made directly by people. This is a highly subjective assessment in nature and is not simply influenced by internal objective parameters (Esteban-Millat et al., 2014). The most appropriate evaluator of QoE is the end user, whose perception of the QoE of a film can be measured by SVQA (Shahid et al., 2014). It is not easy to measure the QoE, which is interfered by many factors. SVQA based on the judgment of observers after careful planning is the most accurate way to measure the QoE (Sedano et al., 2014). When facing the audience outside the laboratory, the slight difference in video quality parameters of different quality films may lose significance due to the influence of environment or hardware. Only the perception of very obvious difference in SVQA is valuable (Sullivan et al., 2008). Considering the importance of SVQA as a part of QoE, this study used the method of controlling OVQA parameters of video samples to investigate and evaluate SVQA of audience.

### 2.8 Satisfaction

Satisfaction is the gap between what consumers expect and what they actually feel about a product or service (D. Choi, Chung and Young, 2019). Audiences make judgments according to advertisements based on their own experience beforehand, and use them as standards to compare with actual experience after watching the advertisements. This particular focal aspect reaction and comparison in a particular time forms satisfaction, which is a cognitive process based on understanding (Brandtner et al., 2021). In addition, emotion is also a component of satisfaction (Palací et al., 2019). The audience's subconscious and automatic emotional experience will affect their satisfaction. Psychological activities, mental states, and emotional feedback from social factors may all influence satisfaction (Yeh et al., 2019). Therefore, there is a close relationship between satisfaction and consumer behavior, and it may also be one of the important factors affecting audiences' feelings towards mobile interactive movie advertisements.

### 2.9 Purchase intention

Purchase intention refers to a person's conscious purchase of a brand, which can be regarded as a consumer's commitment to themselves, corresponding to the likelihood of purchasing a specified product next time (Khuong and Nguyen, 2015). Purchase intention is the likelihood that consumers will plan or be willing to purchase a product or service in the future (P. C. Wu, Yeh and Hsiao, 2011). Higher purchase intention means higher possibility of purchase, but it does not necessarily lead to purchase behavior, and lower intention does not mean that purchase is completely impossible (Y.-H. Wang, Chen and Chen, 2016). Consumers' purchase intention comes from their perception of benefits and value acquisition.

### 2.10 Positive theme

The theme of the video is the topic referred to in the video (Zhu et al., 2020). Themes represent the scope and category of video content. Video as a marketing tool can help consumers imagine products in a more appropriate way (Orús et al., 2017), and increase their purchase intention in a more convincing way (Flavián et al., 2017). As a kind of narrative means, story-oriented video advertisements need to define the theme in the process of recommending products. Audiences tend to see videos with clear themes, which tend to be perceived as high quality (Ham and Lee, 2020). The theme of the video can be divided into positive theme and negative theme according to the audience's feelings. For example, videos featuring good wishes and encouragement made to fight COVID-19 are considered positive types (Buitrago and Martín-García, 2021). There are also videos of COVID-19 related drugs, medical facilities and deaths, which may increase public anxiety and can be regarded as negative types (Al-Zaman, 2021). Therefore, the theme positivity may affect the audience's feelings and evaluation of the advertisement in the process of watching, and further affect consumer behavior.

### 2.11 Acceptance of new media

The concept of acceptance of new media is derived from the technology acceptance model (TAM) to measure the user's adaptation to new technology and the use of psychology (J.-H. Lee, 2021). In addition to TAM, the unified theory of acceptance and use of technology (UTAUT2) is widely used and has attracted attention. These models are developed to explain the user's acceptance of a new technology from the perspective of usage behavior (Ma et al., 2021). In order to use new technologies, media may need to overcome financial constraints, health barriers, and insufficient operational guidance and experience (D. Liu, Liu and Tu, 2020), which means that audiences have to pay extra costs in order to use new forms of media that are iterated by technology. Take interactive video as an example, new media technologies provide more interactivity between audiences and media (Tefertiller, 2020). Different from the traditional media, the role of the audience has been changed and new requirements have been put forward for their adaptability. Therefore, the acceptance of new media may be one of the factors that need to be paid attention to in the design of interactive video advertising.

## 3 Methodology

This study was conducted according to the guidelines of the Declaration of Helsinki and received academic ethics review and approval from the review committee of the Ministry of Social Science, Changshu Institute of Technology. Our experiments informed consent was obtained from all participants and all methods were performed per relevant guidelines and regulations.

### 3.1 Study 1- qualitative exploration of theoretical basis

In study 1, we interviewed respectively seven experts from the academic and industrial fields of interaction, screen imaging and advertising. The background information of the experts is shown in Table 1. The interview included the definition of mobile interactive video advertisements, the age range of the main audience, the important research constructs of mobile interactive video advertisements that need to be discussed, how to evaluate the constructs, the advantages and disadvantages of mobile interactive video advertisements and the future trend, etc. According to the content of expert interview results, a total of 7 interview audio files were collected, with a total length of about 9 h, and textual formation into nearly 31,000 words of verbatim manuscript. Open coding and axial coding for the interview data were carried out, and a list of important research constructs were put forward. After the list of important constructs were obtained, this study adopted the method of selective coding to explore the relationship among constructs.

**Table 1:** Information of interview experts.

Interviewee	Experience	Position	Expertise and work
Expert A	5 years	Deputy department manager	Mobile phone imaging quality monitoring
Expert B	5 years	Senior manager	Mobile products and technologies
Expert C	5 years	Co-founder	Manufacture of new media interactive devices
Expert D	10 years	Associate researcher	Video art research
Expert E	10 years	Account Director	Advertising
Expert F	15 years	General manager	Development of digital interactive products
Expert G	20 years	Associate professor	Screen imaging

Open coding is divided into conceptualization stage and categorization stage. In the process of conceptualization, researchers will not only break up sentences with complete narrative meaning and re-name the concept, but also invite three coders, who worked in advertising, new media industries as coders, to analyze the data of the words together from multiple point of view, and all coding results were tested to verify conceptual reliability (Flick, 2010). In the categorization process of open coding, the conceptual results were logically sorted and merged, and some concepts can be grouped into higher-level concepts to form a higher level (Strauss and Corbin, 1990). Further, each category after the group was given a new tag name to include the concept set to which it belongs.

After entering the axial coding step, the classification results are divided into primary category and sub-category according to the interpretation relationship. According to the definition of principal axial coding by Strauss and Corbin, in this study, each primary category represents a phenomenon respectively. This phenomenon has great significance or strong power to explain. On the other hand, the sub-category does not represent the phenomenon itself, but answers the questions about the phenomenon such as when, where, why, who, how and with what consequences. Sub-categories can give more explanatory power to primary categories.

Selective coding returns to the original interview with the participation of each main category through the series of core categories. Analysis of the memo helps to select a core category from all the main categories as a guide for theory construction and data collection (Strauss, 1987), and other codes are subject to the focus of the core category. Through the concatenation of the core categories and the participation of each main category, the relationship between each construct mentioned in the original text and other constructs was found, the result of the encoding construct was outlined, and the relationship model was established. After establishing the dimension relationship through selective coding, this study also examines whether there is a logical gap, which makes up for the imperfect category development and the category pruning that covers too much data, and finally proposes the hypothesis model.

In addition, in order to develop specific sacale for mobile interactive video advertising, this study conducted review of the axial coding of each construct, the open code under the classification of axial coding were found, and then the original verbatim manuscript of the interview content was used as reference to obtain the original items. The item bank will be expanded by combining the newly added items and the items collected in the literature review. The expert focus group meeting 1 was held to confirmed the content validity of each construct. The background information of experts is shown in Table 2.

**Table 2:** Expert focus group 1 information.

Participants	Seniority	Position	Specialty and work items
ExpertsA	3 years	Associate Professor	Color research
ExpertsB	10 years	Lecturer	Animation creation
ExpertsC	20 years	Assistant professor	Media art

### 3.2 Study 2- production of research samples

In this research stage, 5 experts from interactive and video majors were invited to form a focus group 2. The information of the experts shown in Table 3. This study collected mobile interactive video advertising from the market as much as possible. Expert focus group 2 selected the sample story based on the relatively moderate evaluation of the content and theme from the collected samples. Brand information was hidden in the sample remake process. In this way, variable control is realized in this study.

**Table 3:** Focus group 2 expert information.

Participants	Seniority	Position	Specialty and work items
ExpertsA	5 years	Producer	Video production
ExpertsB	10 years	Interaction designer	Interaction design
ExpertsC	20 years	Assistant professor	Media art
ExpertsD	10 years	Lecturer	Animation creation
ExpertsE	10 years	Lecturer	Interaction design

In addition, the content to be made should be determined based on the results of the division of product involvement and interactive narrative when making the research samples. Previous studies have shown that interactive narrative is composed of “Structure of Interactive Nodes”, “Mode of user participation” (Ryan, 2006). Therefore, the focus of division is to judge how many types of text structure and interactive form there are respectively. This study divided the categories of product involvement degree (high/low), text structure and interactive form, and recombined them to make research samples.

In order to select the products with the highest and lowest product involvement, this study summarized the collected product types involved in mobile interactive video advertising. After the duplicates are removed, the remaining product types are converted to the form of graphics cards. Respondents with characteristics consistent with the main audience group were invited to regroup the product cards in an unlimited number of groups, and the number of cards within each group was also unlimited. This offline survey was conducted in October 2018. 30 respondents aged 15–29 years were tested, including 15 males and 15 females. After the completion of the survey, the data were analyzed by means of multidimensional scaling and cluster analysis, and the sample groups were divided. Representative samples of each group were selected by expert focus group 2.

After that, another offline questionnaire survey was conducted in November 2018. A total of 60 males aged 15 and 29 years were invited, including 30 males and 30 females. Participants were asked to rate the degree of product involvement of each representative sample. The 12-item product involvement scale was used to measure the level of product involvement of people in line with the main audience groups. The questionnaire scale is shown in Table 4. If the measurement results are significantly different between the highest and lowest products, the product involvement degree is differentiated.

**Table 4:** Scale of product involvement.

Items	Source
1. This product tells other people sth. about me.	(Bauer et al., 2006)
2. It helps me express my personality.	
3. It does not reflect my personality.∗	
4. It is part of my self-image.	
5. It is not relevant to me.∗	
6. It does not matter to me.∗	
7. It is of no concern to me.∗	
8. It is important to me.	
9. This product is fun.	
10. I find it fascinating.	
11. I find it exciting.	
12. I am interested in it.	

∗ Item is reverse scored.

In order to distinguish the interactive narrative, this study collects and organizes the types of text structure and interactive form respectively. In the part of text structure, a total of 30 different types are collected through literature review. In terms of interactive forms, this study looked at the industry's existing examples of interactive video advertising and found 10 different types of interaction. After that, representative samples were selected after discussion in expert focus group 2.

### 3.3 Study 3- quantive validation of the hypothesis model

In Study 3, the structural equation model was used to quantitatively verify the hypothesis model proposed in Study 1. Participants were invited to watch the mobile interactive video advertisement sample produced in Study 2 and fill in the questionnaire. The watching environment was rigorously designed in this study. The 6500K color temperature kinyo removable LED metal lamp (PLED-422) was selected as the light source. Firstly, the brightness and contrast are adjusted subjectively after using the prescribed debugging signal input monitors produced by the standard (ITU-R., 2018). The illumination conditions of the site and facilities were measured with TES-1330A2 million Lux digital luminometer. The results show that the ratio of luminance to peak luminance of the inactive tube screen is approximately 0.01 (less than or equal to 0.02), the ratio of luminance of the screen showing only the black level to the corresponding screen showing only the peak white level in a completely dark room is approximately equal to 0.03 (approximately equal to 0.01), the brightness and contrast of the display are adjusted subjectively by the Pluge signal, the maximum observation Angle relative to the normal in the interaction process is not more than 30°, the ratio of the background brightness behind the image monitor to the peak brightness of the image is 0.16 (approximately equal to 0.15), and the indoor light source is a low-lit D65 light source. In summary, the lighting of the site and facilities met the recommended requirements (ITU-R., 2012).

Through the pre-test, the questions with unclear meaning were recorded, and the researchers further introduced and explained these questions to the subjects, observed which expression was easier for the subjects to understand, and modified the expression of the questions in a way that was easier for the subjects to understand. This phase of the survey was conducted offline in April 2019. According to the definition of the characteristics of the main audience, 20 subjects, including 10 males and 10 females, were invited to conduct a pre-test study.

As the survey officially started, according to the definition of the characteristics of the main audience, 100 subjects were recruited in northern Taiwan strictly according to the gender division of 50 males and 50 females. This phase of the survey was conducted offline from May to July 2019. The respondents were asked to watch all the samples one by one in a random order in the field set up for this study, and the length of each sample was about 1 min. During the watching process, the respondents were allowed to interact with the mobile interactive video advertisements samples according to their own wishes, which includes operating with the interactive form provided by the advertisements, and choosing the direction of the story within the structure of the text established by the current sample. After watching, the respondents were asked to fill out a questionnaire about the current sample before watching the next sample. Participants were paid 400NT each to take part in the test.

## 4 Results

### 4.1 Study 1- expert interview and interview data analysis

#### 4.1.1 Age definition of primary users

Seven experts judged the age of the main audience of mobile interactive video advertisements, including 6 times of audiences under the age of 19, 5 times of audiences between the age of 20 and 24, 6 times of audiences between the age of 25 and 29, and all the other age ranges were selected less than 2 times. According to a statistical report on big data of digital netizens in Taiwan released by the survey institute, the number of Internet users between the ages of 6 and 14 in Taiwan is only 5.9% compared with the overall number of Internet users (InsightXplorer, 2018), and this part of the Internet users is very small. Therefore, when judging and dividing the main audience groups of mobile interactive video advertisements, the importance of audience groups in this part of age range should be reduced. Based on the experts' opinions on the age range of the main mobile phone audience and the literature, this study considers that the 15–29 years old group is the main audience of mobile phone interactive video advertising, and chooses them as the research object.

#### 4.1.2 Open coding

Qualitative textual analysis data is based on the results of interviews with experts and coded according to Corbin (2021) suggestions for text coding methods. In the conceptualization stage, a total of 263 basic concepts were encoded, and these concepts appeared 1176 times. To verify the consistency of the coding, in this study, Krippendorff α was used to test the conceptual results of triangulation (Lombard et al., 2002). In the process of calculation, data were analyzed based on SPSS macro instructions (Hayes and Krippendorff, 2007). In this study, the SPS-based macro instruction of Hayes and Krippendorff was adopted for qualitative data analysis (Hayes and Krippendorff, 2007). After entering the macro command, execute the command to activate the macro command:

kα judges = obsa obsb obsc obsd/level = 2/detail = 1/boot = 10000.

Among them, obsa, obsb, obsc and obsd represent the conceptual results of researchers and other three coders in the open coding stage of this study. Level = 2 means that data is calculated on a sequential scale; Detail = 1 means that all computational details will appear in the result; Boot = 10000 indicates a certain number of boot iterations in the operation. According to Krippendorff's suggestion, it is customary to require α ≥ .80 for the result to be reliable (Krippendorff, 2004). In this study, the final fitting value of Krippendorff α was.82, indicating that the conceptual results of this study were relatively reasonable when compared with the conceptual results of 3 coders and passed the reliability test.

#### 4.1.3 Axial coding

During the axial coding process, 74 results were classified into the main categories and the sub-categories according to breadth of the concept. The primary and secondary categories are related to each other along a logical relationship of attributes and planes. The main categories proposed by this study include: content desirability, brand awareness, product involvement, interactive narrative, immersion, QoE, satisfaction, purchase intention, positive theme, and acceptance of new media. The remaining categories are sub-categories used to interpret the 10 main categories. The relationship between the main categories and the sub-categories is as Table 5 shows.

**Table 5:** Axial coding results.

Main category	Sub-category	Main category	Sub-category
Content desirability	The ornamental value of advertising, the quality of costumes and props, unexpected elements in the watching process, story, dramatic tension, the performance of the actors, The attractive elements of advertising, pros and cons of the video, editing and packaging, shooting quality of picture, understandability, audience's willingness to try, the appeal of advertising	Brand awareness	Brand style, the spreading power of advertising, motivation to watch, communication elements, CTR (click-through rate)
Product involvement	To hide irrelevant marketing	Interactive narrative	The level of audience interaction, new perspective offered by advertising, plausibility of plot and interaction, meaningfulness of interaction, interactive games with moderate difficulty, text structure, manipulation of the action, interactive technology, advertisement with interactivity, acceptance of audience, to provide audience with different sensory feelings, diverse interactive experiences, audience's influence on advertisements
Immersion	Immersive, spiritual experience, audience's attention on watching, elements of mystery, full screen, humer experience	QoE	Aesthetic perception, tonal, image quality, carrier features, watching place and situation
Satisfaction	The amount of time the audience wants to watch, to praise the audience in a more grandiose way, innovation of the advertisements, artistry, the story flows smoothly as when watching, the spread among audience, attention to details, appropriate advertisements length, guidance of interaction, user experience of advertising, design quality of advertising, relax to watch	Purchase intention	Advertising appeal、target consumer groups
Positive theme	Positive emotional acquisition, the emotional resonance of the audience to the advertisement, degree of touching	Acceptance of new media	Production cost, capability of advertising producer, advertising features

#### 4.1.4 Selective coding

The way of writing the research review story line helps to determine the core category purchase intention. This core category is sufficient to illustrate the connotation of the whole qualitative data analysis process: “Enhancing purchase intention effect through mobile interactive video advertising”. In these interviews, what struck us again and again was that various concepts surrounding the audience's judgment on the quality of mobile interactive video advertisements kept appearing, while the number of concepts about the final commercial effect was relatively small. Some people think that mobile phone interactive video advertising is an art form, is a work of art, but its essence is still advertising, advertising is to explore whether the effect can be produced, the effect is good, which is the primary problem to be solved as an advertising. Through watching mobile interactive video advertisements, the target population has generated various judgments about mobile interactive video advertisements, which should ultimately correspond to whether they can enhance their purchase intention and bring good commercial effects. The unstated but objective possibility is that each major category will have an impact on the purchase intention, but the size of such an impact needs to be verified.

Back to the text data, find the discussion on the influence relationship between main categories from the text context, so as to prove the path relationship between main categories. Word for word analysis results show that there are 30 affected paths in total. This study lists the original content of each relationship path as supporting data, as shown in Table 6. Among all path relationships, content desirability degree has affected other main categories most times, pointing to other main categories 7 times in total. The main categories that were affected the most times were purchase intention, which were pointed to by other main categories for 6 times in total. As the construct of purchase intention shows a very high probability of being affected, the result of data review further verifies the accuracy of selecting the core category as purchase intention in the previous step.

**Table 6:** Relation link result and interview text basis.

Relationship	Basis	Relationship	Basis
1.Content desirability - > Product involvement	*A good story will increase product involvement*	16.Interactive narrative - > Purchase intention	*Consumers really want interaction when they watch advertisements in order to buy a product*
2.Content desirability - > Interactive narrative	*People who are interested in the topic or content will be easily attracted by the interaction*	17.Immersion - > Content desirability	*Interactive films have a stronger sense of substitution, thus increasing the user's favorable impression of the video content*
3.Content desirability - > Immersion	*Recognition of content will be an indicator of immersion*	18.Immersion - > Product involvement	*The engagement degree of interactive products is affected by the immersion degree of experiencers*
4.Content desirability - > QoE	*Traditional measures should not be abandoned because of the inclusion of interaction, for example, content will also greatly affect the quality of the film*	19.Immersion - > Interactive narrative	*User immersion and substitution are the basic factors affecting interaction*
5.Content desirability - > Satisfaction	*Even if there is no interaction in the video, good content can still make audiences feel satisfied*	20.QoE - > Content desirability	*Users decide whether to continue watching based on the first impression of the picture*
6.Content desirability - > Purchase intention	*It can be seen from the audience's watching duration that the good content can support audience's consumption.*	21.QoE - > Immersion	*Achieving an immersive experience requires good picture quality, and color is also important*
7.Content desirability - > Positive theme	*If the audience think it is made up after watching the short film, it is impossible to bring emotional resonance to the theme*.	22.QoE - > Satisfaction	*Satisfaction with the video is composed of product involvement, quality perception and interaction form*
8.Brand awareness - > Content desirability	*Brand endorsement helps increase consumers' interest.*	23.QoE - > Purchase intention	*Smaller pictures make people less interested in advertising*
9.Brand awareness - > Purchase intention	*In terms of purchasing a mobile phone, brand is more important than advertising promotion.*	24.QoE - > Positive theme	*Low-quality videos may not resonate with audience*
10.Product involvement - > Satisfaction	*Satisfaction with the video is composed of product involvement, quality perception and interaction form*.	25.Purchase intention - > Content Desirability	*If the topic of the advertisement is easy to understand and the consumer is interested in it, he/she will continue to watch it.*
11.Product involvement - > Purchase intention	*The relationship between the product and audience can tell how good the product is and necessity to buy it.*	26. Positive theme - > Immersion	*The resonance of the theme is also the factor that causes immersion.*
12.Interactive narrative - > Product involvement	*Different interactions lead to different levels of product involvement and quality perceptions.*	27. Positive theme - > QoE	*Video quality is influenced by topics such as art, reality, emotion, cognition, aesthetics, history, etc.*
13.Interactive narrative - > Immersion	*The lowest level of interaction is the presence or absence, good interaction design can help reach immersion.*	28.Positive theme - > Satisfaction	*The audience's feelings about the topic of art and emotion should affect satisfaction*.
14.Interactive narrative - > QoE	*Different interactions lead to different levels of product involvement and quality perceptions.*	29.Acceptance of new media - > Content desirability	*New forms of media are more attractive to audience.*
15.Interactive narrative - > Satisfaction	*The first is the interactive experience, the second is the quality of the film... Satisfaction is up to these two factors.*	30.Acceptance of new media - > Purchase intention	*If this media is targeted for advertising use, I think it has a share in future marketing, in a form that consumers can accept and induce purchase behavior*

#### 4.1.5 Establishment of hypothesis model

**Figure 3:**
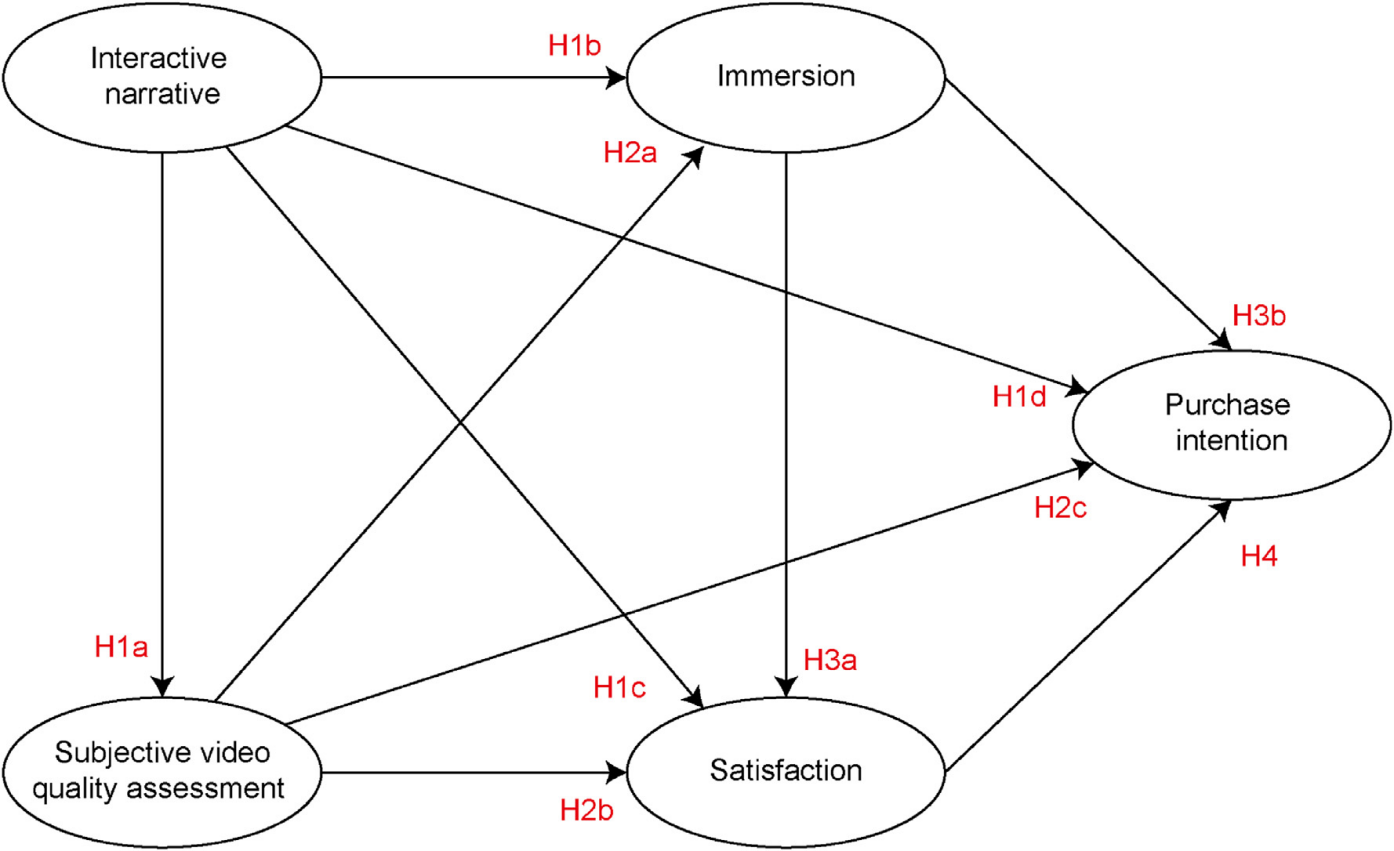
Research hypotheses.

Based on the model obtained from qualitative research, this study simplifies the path relationship. Furthermore, the possible influence paths are supplemented according to the dimension relationships in the literature, and the hypothesis model is finally proposed (See Figure 3). Considering that content desirability, brand awareness, positive theme, acceptance of new media and other aspects are included in the model, it is necessary to make and provide multiple samples to the respondents so that they have enough experience to evaluate. This can lead to a multiplicity of studies in scale and time. Therefore, this study takes these four dimensions as control variables and measures them in more depth in the subsequent research. Product involvement is treated as a moderating variable in the model, and this research will examine its moderating effect in each of the influence paths. Based on the coding results and literature, the following hypotheses are proposed.

In order for customers to get better QoE, interactive narrative should be paid attention to in the process of advertising design (Katifori et al., 2018). Interactive narrative can make the quality of the story perceived by the experiencer higher (Rao et al., 2019). The more users enjoy interactive narrative, the higher QoE they will feel (Swanson and Gordon, 2010). A more reasonable and rich interactive narrative improves users' QoE (Santiago et al., 2014). Subjective Quality assessment is the result of perception of QoE, so there may be a causal relationship between customers' perception of interactive narrative and SVQA.

On the other hand, previous studies have shown a relationship between interactive narrative and immersion. If the audience is satisfied with the interactive narrative, they can better resonate with the narrator and understand the content of the story in an immersive way (Rizvic et al., 2016). For example, using an interactive narrative of a news story can make the audience feel immersed (Domínguez-Martín, 2015). When customers participated in interactive narrative's ads, they might have gotten hooked and become immersed in the story (Ching et al., 2013).

In addition, a nature education study of elementary school students showed that telling stories in an interactive narrative style led to higher satisfaction (Weng et al., 2011). Compared to linear Narrative, interactive narrative can make users feel satisfaction (De Lima et al., 2014; de Lima et al., 2012). During the online education process, interactive narrative provides an immersive way to motivate learners to present the course content and ultimately help students achieve higher satisfaction (Baldwin and Ching, 2017). Since satisfaction often influences purchase intention in the marketing process (Moslehpour et al., 2018), interactive narrative may influence both satisfaction and purchase intention. For example, when virtual reality is used in marketing, the narrative it provides as an interactive medium enables customers to have higher purchase intention (De Regt, Plangger and Barnes, 2021). Interactive narrative provides the possibility to improve customers' purchase intention from the perspective of advertising design. Augmented reality, as an interactive medium, is often used for storytelling of cultural creativity and can improve purchase intention (Verhulst et al., 2021). Based on literature and path relationship obtained from qualitative research, this study further proposed hypotheses:

Hypothesis 1 (H1): Interactive narrative has a positive influence on (H1a) SVQA; (H1b) immersion; (H1c) satisfaction; and (H1d) purchase intention.

Previous studies have shown that customer QoE is often associated with immersion. For example, in the experience of virtual display effects, customers' evaluation of advertising quality may have an impact on immersion through the mediation of other variables (M. Lee, Lee et al., 2020). There may be a correlation between the quality of augmented reality and immersion (Cummings and Bailenson, 2016), This suggests that higher advertising quality may inspire immersion in the customer.

Many studies show that there is a correlation between quality and satisfaction. Quality is an important factor influencing satisfaction (Martensen et al., 2000). Satisfaction can be measured by customers' judgment of quality (Mouri, 2005), and the process of quality judgment always happens before satisfaction (Taylor et al., 1993).

Many studies have shown that purchase intention is also closely correlated with quality (Dash et al., 2021). For example, customers take the quality of advertisement into full consideration when making a purchase decision (Kim et al., 2020). At the same time, there is evidence that customers' QoE in advertising affects satisfaction and purchase intention when purchasing products (Tsiotsou, 2006). SVQA is one of the important means to evaluate QoE. Customers' perception of advertising SVQA may directly affect immersion, satisfaction and purchase intention (Zarei et al., 2019). Based on literature and path relationship obtained from qualitative research, this study further proposed hypotheses:

Hypothesis 2 (H2): SVQA has a positive influence on (H2a) immersion; (H2b) satisfaction; and (H2c) purchase intention.

Considering the important role of immersion in multiple media field models, it is necessary to discuss the relationship between immersion and customer satisfaction and purchase intention. Previous studies have shown that more immersive experiences lead to higher satisfaction (Cummings and Bailenson, 2016; Reysen et al., 2019). Media can help improve user satisfaction to some extent by improving immersion (Xi and Hamari, 2019). The enhancement of audience immersion will have a positive effect on video satisfaction (Fornerino et al., 2008).

Similarly, the association between immersion and purchase intention is illustrated in many literatures. For example, the research of interactive advertising on shopping websites shows that customer immersion has a positive impact on purchase intention (Biocca et al., 2001). More immersion shopping experience will promote customers' purchase intention (Y.-S. Wang et al., 2019). Immersion will make the user feel more realistic, further triggering the purchase behavior (S.-L. Wu and Hsu, 2018). Therefore, this study further proposed the hypothesis:

Hypothesis 3 (H3): Immersion has a positive influence on (H3a) satisfaction; and (H3b) purchase intention.

Previous studies have shown that customer satisfaction positively affects behavioral intention (Ali et al., 2016; Hosany and Witham, 2010; tom Dieck et al., 2017). Satisfaction has become an important part of marketing, and customer satisfaction leads to purchase intention (Ball et al., 2004). Customer satisfaction is considered as an important indicator of purchase intention (Reichheld and Teal, 1996). This shows that overall satisfaction of advertisements is very important. Except for video advertisements, satisfaction also shows a close relationship with purchase intention in other kinds of advertisements. For example, research on the promotion methods of travel agency websites shows that website satisfaction has a positive impact on customers' purchase intention (Hsu et al., 2012). A test of customers viewing mobile ads using augmented reality technology showed a significant correlation between satisfaction and purchase intention (Sung, 2021). Therefore, this study further proposed the hypothesis:

Hypothesis 4 (H4): Satisfaction has a positive influence on purchase intention.

#### 4.1.6 Development of questionnaire

Through qualitative coding, a total of 44 original items were obtained from the qualitative data analysis of the 5 constructs. The acquisition of questions starts from the original interview, goes through the conceptualization and categorization coding in the open coding stage, and further classifies in the axial coding stage, and finally extracts the relevant questions. Moreover, this study also collected the existing items of various dimensions in the literature and added them to the total items, and the items with similar meanings were finally merged.

The questionnaire with content validity was obtained after the items edited and deleted by expert focus group 1. The results indicate that the remaining 7 items in the interactive narrative were all developed in study 1 stage. The remaining 6 items in the SQVA, 2 of which are from the literature (Kortum and Sullivan, 2010), and 4 of which are developed in study 1 stage. There are 7 remaining items in the immersion, 4 of which are from the literature (Jennett et al., 2008), and 3 developed in study 1 stage. The remaining 6 items in the satisfaction were all developed in study 1 stage. The remaining 6 items in the purchase intention are all from the literature (Bian and Forsythe, 2012; Kwon et al., 2007; Li et al., 2002; Michaelidou and Hassan, 2008; Moon et al., 2008). The complete questionnaire questions used for the survey can be found in the supplementary material entitled Structural Equation Modeling Questionnaire.

### 4.2 Study 2- results of research sample production

#### 4.2.1 Classification of product involvement

**Figure 4:**
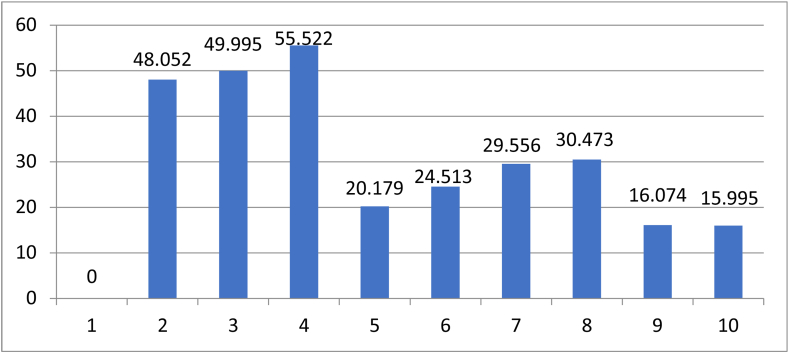
Incremental percentage of data coefficient of the last ten strokes.

After the same item is removed from the product categories of the above collected samples, 27 product categories are obtained, including game APP, bank, etc. Respondents’ perception of the clustering outcome between each product category was converted from the same number of times to different times. The higher the number of times, the more subjects believed that the two product categories should belong to different groups. Multidimensional scaling was carried out for Euclidean straight-line distance results of each product category, and six dimensions were selected for numerical calculation. The results show that Stress = 0.034 is less than 0.05, and R^2^ (RSQ) = 0.985 is greater than 0.5, indicating good fitting. The results of multidimensional scaling analysis were passed into cluster analysis to be calculated. Wards Method was recruited to analyse without specifying the number of clusters. It turned out that through to the group of 20% of the data after the condensation process, namely 21–26 times condensation process of observation, which did not appear before the stage has not yet been condensed or agglomerate item in earlier stage, so the numerical value does not appear in the process of condensation outliers. In this study, the data of the last 10 times of coagulation were taken, arranged in reverse order and the incremental percentage of coefficients was made into a bar graph, as shown in Figure 4. The 1 represents the final condensation, which means there is no condensation later to be used in the calculation of the incremental percentage. Through the observation of the increment percentage of the data coefficients of the last 10 data, it is found that there is an obvious increment percentage decrease in the fourth to the last and eighth to the last aggregation, so the number of clusters is preliminarily set as 4 or 8 groups.

The Ward method of hierarchical cluster analysis was used to analyze the results of six-dimensional scale again, and the number of clusters was designated as 4 groups and 8 groups respectively. Due to the obvious differences between the appropriate clusters, this study used univariate analysis of variance to test the results of 4 and 8 groups of hierarchical cluster analysis respectively. The results showed that the variances of the four groups were significantly different among the four groups, and the variances fluctuated too much and the results of the analysis of variance between the four groups were not significantly different. Therefore, the results of the four groups were not appropriate in the analysis of the class cluster. On the other hand, the hierarchical cluster analysis results of the specified 8 groups all passed the standard, so this study used the designated 8 groups as the clustering basis. The appropriateness test results of clustering are shown in Table 7.

**Table 7:** Appropriateness test results of clustering.

Specifies the number of clusters	Dimension	Levene test		ANOVA	
		statistic	Sig.	F	Sig.
4 Groups	1	.378	.770	163.909	.000∗
	2	2.851	.060	227.902	.000∗
	3	.526	.669	94.265	.000∗
	4	.888	.462	.045	.987
	5	3.178	.043	.243	.866
	6	.984	.418	.052	.984
8 Groups	1	2.411	.067	175.018	.000∗
	2	2.055	.108	146.131	.000∗
	3	0.608	.721	39.926	.000∗
	4	1.657	.186	4.464	.004∗
	5	1.622	.195	6.985	.000∗
	6	0.476	.818	3.172	.021∗

∗ The level of significance is 0.05.

Further, K-means cluster analysis is used to verify the clustering results of the 8 groups specified in the hierarchical cluster analysis. Ten times of iteration were selected, and the mobile average was used for analysis. The number of clusters was designated as 8 groups. Kappa comparative analysis was conducted between the clustering results and those of the 8 groups designated by hierarchical cluster analysis. The results showed that the value of 0.827 was greater than 0.7, and the significance of approximate t-test was less than 0.05, which indicated that the clustering results of the two methods were similar, and the cluster analysis results were reliable. To sum up, this study used the hierarchical cluster analysis of 8 groups to cluster 27 product categories from 40 samples collected, and selected the representative samples of each group to test the product involvement degree. The results of clustering and representative sample selection are shown in Table 8.

**Table 8:** Product division and representative product selection results.

Groups	Product category				
	Representative sample	Other samples			
1	PC game	Music software	Mobile phone games	Mobile phone input method	
2	Search Engine APP	Browser APP	Takeout platform APP	Video APP	Take a taxi APP
3	Retail stores	Transportation bureau	Bank		
4	Drinks	Cleanser	Dress	Diamond	
5	E-commerce platform	News website	Live platform	Fundraising platform	
6	Mobile phone	Car	Electrical appliances		
7	Artificial intelligence company	Advertising company	Game company		
8	Fatigue driving detection system				

Kruskal-Wallis ANOVA was used to test the evaluation results, and the significance of the results was less than.05, indicating that there were significant differences in the level of involvement of at least two groups of products in the 8 categories measured. Among them, mobile phone has the highest product involvement degree with an average of 4.21, while fatigue driving detection system has the lowest product involvement degree with an average of 2.95. Therefore, we chose mobile phones and fatigue driving detection system as representative products with high and low product involvement.

#### 4.2.2 Classification of interactive narrative

**Figure 5:**
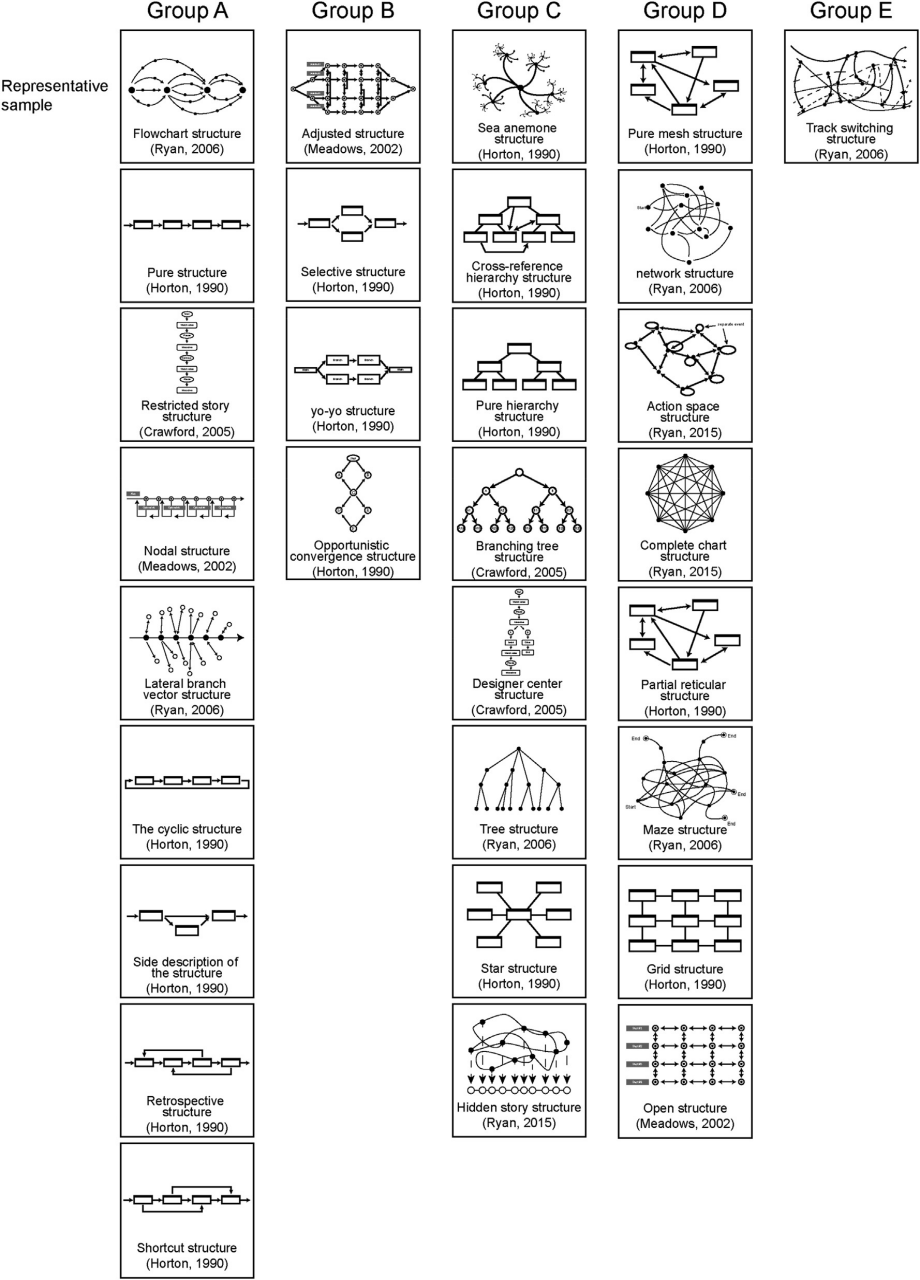
Text structure grouping and selection (Crawford, 2005; Horton, 1990; Meadows, 2002; Ryan, 2006, 2015).

Text Structure was divided into 5 categories according to the discussion of expert focus group 2 (See Figure 5). The representative sample in each category was selected through experts’ voting. The result showed that “Flowchart Structure” was chosen three times in category A and got the highest number of votes. Category B has the highest number of occurrences of “Adjusted structure”, which was selected twice. Category C appeared the most: “Sea Anemone Structure” appeared for 3 times; Category D appears most frequently with “maze text architecture”, “Pure Mesh Structure” and “Complete Chart Structure” appearing twice each. Due to the same number of votes, the experts jointly chose “Pure Mesh Structure” as the representative sample through negotiation. Category E has only one sample “Track Switching Structure” and is therefore automatically selected.

**Figure 6:**
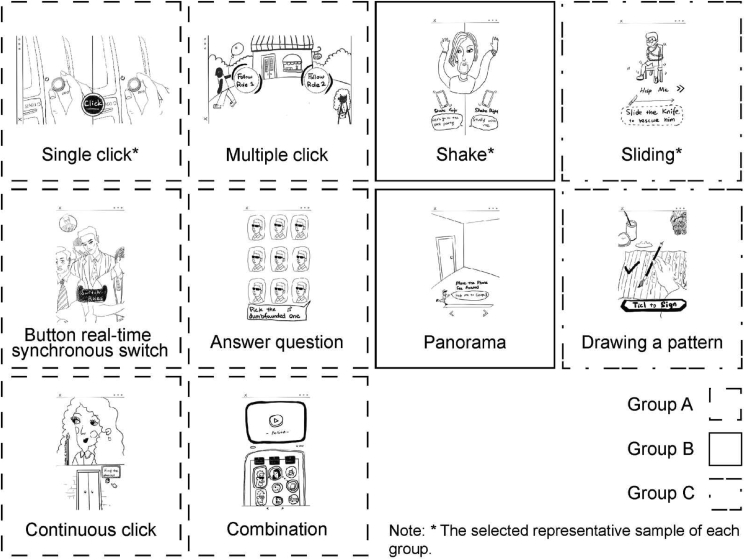
Interactive form grouping and selection.

After the further discussion of expert focus group 2, these interactive forms are divided into three categories according to operation. The interaction forms of category A include “button real-time synchronous switch”, “continuous click”, “single click”, “multiple click”, “answer question” and “combination”, and the representative sample is “single click”. The interaction forms of category B include “shake” and “panorama”, and the representative sample is “shake”. The interaction forms of category C include “sliding”, “drawing a pattern”, and the representative sample is “sliding”. The clustering results of the interactive form are shown in Figure 6.

By combining the classification results of text structure and interactive form, this study produced new samples for mobile phone and fatique driving detection system. Each product has a total of 15 samples of 5 text structure combinations and 3 interactive forms, and a total of 30 samples were made.

### 4.3 Study 3- quantification verification of the model hypotheses

#### 4.3.1 Pre-test

The results of pre-test showed that the Cronbach's α coefficients were all above the acceptable level of 0.70 (Santos, 1999), and the reliability of the construct when each item was deleted was not higher than the original construct. Because the test result of the item reaches the standard, this stage will not be deleted.

#### 4.3.2 Unidimensionality test

Firstly, exploratory factor analysis is carried out by principal component analysis. Rotating axis method is the maximum variation method, and new factors with eigenvalues greater than 1 are extracted (Harman, 1976). The results show that the SVQA4 item in the SVQA construct should be deleted because the commonality is less than 0.5 (Carter et al., 2012; Kang and Kim, 2017; Yana et al., 2015). After deletion, the KMO test result of exploratory factor analysis for all constructs was greater than 0.5, and the Bartlett spherical test result was significant (Kaiser, 1974; Norusis, 1998). The commonality of the items in all constructs was greater than 0.5, and the cumulative explanatory variation was 69.9%–86.21%, and only a single factor could be extracted. Confirmatory factor analysis (CFA) was further used to test each dimension separately. The results show that after deleting items IN4, IN6, IN7, IM2, SA1, SA5, PI2 and PI5, the model fitness index of each dimension reaches the standard in CFA test respectively. In conclusion, the data meets the requirement of unidimensionality (Kohli et al., 1998).

#### 4.3.3 Measurement model

First-order confirmatory factor analysis was performed on the whole model, and the results were deleted from large to small according to Modification Indices. After further deleting IN2 items in the interactive narrative, SVQA1 and SVQA6 items in the subjective video quality assessment, IM1, IM4 and IM7 items in the immersion, SA6 items in the satisfaction, and PI6 items in the purchase intention, the model index reached the standard (χ2/df = 4.637, CFI = .929, GFI = .964, NFI = .912, AGFI = .946, RMSEA = .035, SRMR = .034). Therefore, the above items will be deleted. The reference standard of χ2/df has two levels, less than 3 represents a good fitness, and less than 5 indicates a fair fitness (Sutsunbuloglu et al., 2022; Tutar and Avcı, 2022). In addition, CFI, GFI, NFI, AGFI and other indices are greater than 0.9, and RMSEA and SRMR are lower than 0.05, which also meet the model fit criteria suggested in previous studies (S. A. Lee and Neimeyer, 2022). These results of model fitness indices show that the model has a good fitness. After the item is deleted, the combinatorial reliability of each constructs reaches above 0.8, indicating that it has the combinatorial reliability (Hair et al., 2006). The factor load of each observation item was all greater than 0.5, and the average variation extraction (AVE) of each aspect was all above 0.5, indicating convergence validity (Fornell and Larcker, 1981). Confirmatory factor analysis tests are shown in Table 9.

**Table 9:** Confirmatory factor analysis test results of the overall model.

Constructs	Coding	Items	Factor loading	AVE	CR
Interactive narrative	IN1	I think the interaction of the AD is meaningful	.865	.752	.901
	IN3	I think the AD gave me a new perspective	.875		
	IN5	I think the interactive experience provided by this AD is diverse	.862		
Subjective video quality assessment	SVQA2	I think the design of this advertisement is exquisite	.887	.827	.935
	SVQA3	I think the advertisement is aesthetically pleasing	.928		
	SVQA5	I think the colors in this AD are nice	.913		
Immersion	IM3	I'll be interested to see how this advertising thing goes	.880	.773	.911
	IM5	I'm really focused on watching this AD	.899		
	IM6	I think watching this AD is experiencing something rather than just doing something	.858		
Satisfaction	SA2	I think this AD has an appropriate advertising length	.826	.723	.887
	SA3	I think the advertisement is creative	.839		
	SA4	I think this AD gives a good user experience	.885		
Purchase intention	PI1	If I have a need, I'm more likely to buy the advertised product	.942	.877	.955
	PI3	I will buy the product in the advertisement	.932		
	PI4	I will probably buy the advertised product	.936		

Pearson correlation analysis of each construct shows that the square root of the average variation extract of each dimension is greater than the correlation coefficient between it and other constructs, which means any dimension of the model shares more variance with its associated indicators than with any other construct (Hair et al., 2014). Previous studies showed that the potential collinearity between dimensions could be estimated through correlation coefficients, the common standard of correlation coefficient is over 0.8 (Provenzano et al., 2020; Ren et al., 2020; M. Wang et al., 2020). Hence, no potential collinearity problems existed in our dataset. In summary, the expression has discriminative validity (Fornell and Larcker, 1981). The discriminant validity test is shown in Table 10.

**Table 10:** Results of Fornell-Larcker criterion test.

	IN	SVQA	IM	SA	PI
IN	.867				
SVQA	.629∗	.909			
IM	.710∗	.651∗	.879		
SA	.747∗	.720∗	.758∗	.850	
PI	.611∗	.627∗	.727∗	.711∗	.936

∗ The level of significance is 0.05.

#### 4.3.4 Structural model

**Figure 7:**
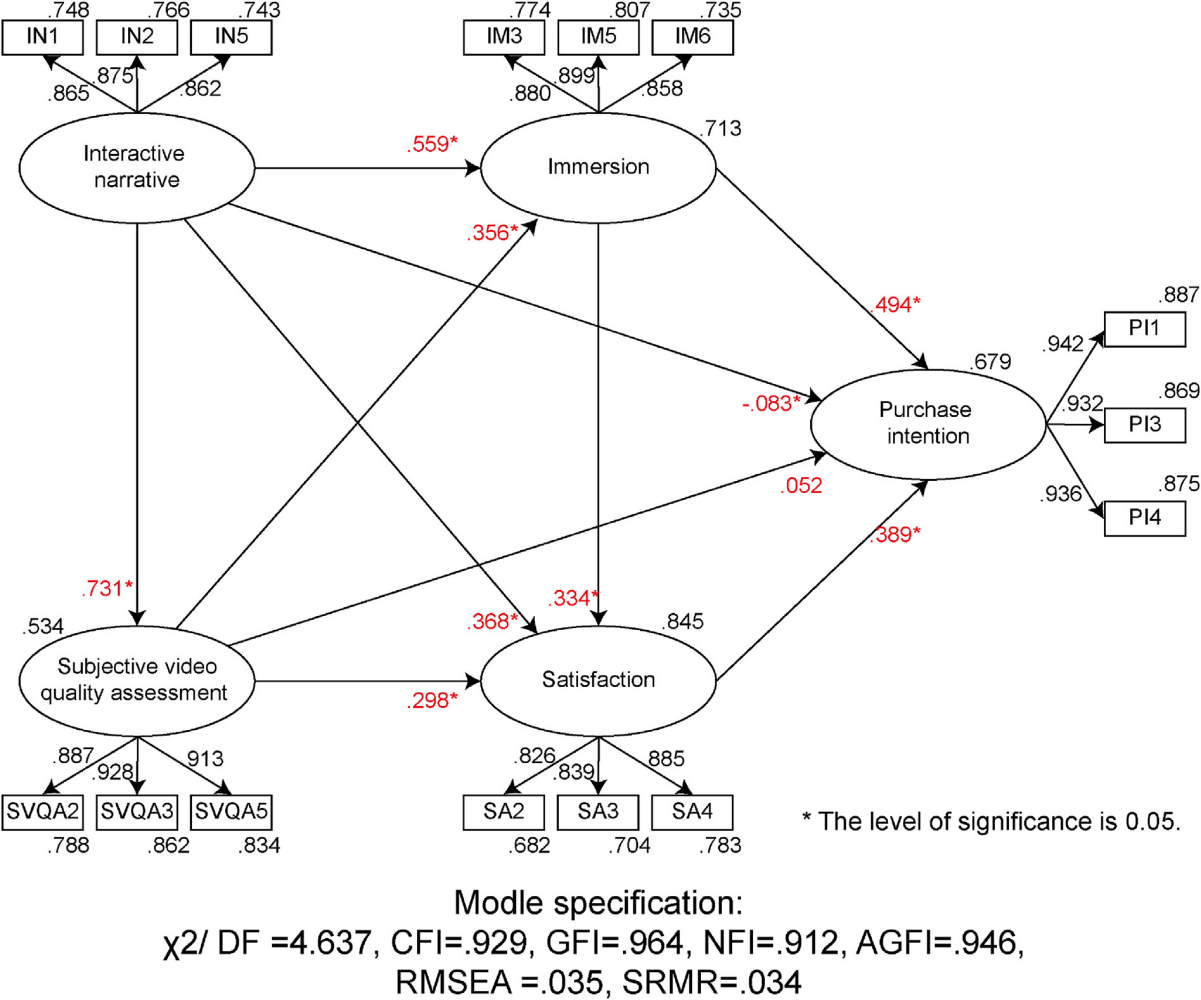
Path analysis results.

Path analysis was performed with AMOS 22 statistical software. In order to test the path coefficient, 2000 times of Bootstrap were used. Since the sample size is greater than 1000, it is suitable for computing with Asymptotically distressing-free method (Browne, 1984). After testing, the indicators of the model reached the standard (Hair, 2009). Results of standardized path analysis were shown in Figure 7.

The path coefficient results are shown in Table 11. Except that SVQA on purchase intention is not significant, the other influence relationships are significant. It is worth noting that the path coefficient of interactive narrative on purchase intention is less than 0.1 and the significance is relatively weak. In the case of large sample size, the significance level can be reached even if the path coefficient is small. From the perspective of effect size, the path coefficient should be taken as the main consideration. Therefore, the causality between the variables is not considered close enough. In summary, H1d and H2c were not supported, while H1a, H1b, H1c, H2a, H2b, H3a, H3b and H4 were all supported.

**Table 11:** Results of path analysis.

Hypothesis	IV	DV	Direct effect		Indirect effect		Total effect		Results
			β	Sig.	β	Sig.	β	Sig.	
H1a	IN	SVQA	.731	.016∗	/	/	.731	.016∗	Support
H1b		IM	.549	.000∗	.260	.006∗	.809	.005∗	Support
H1c		SA	.368	.000∗	.488	.004∗	.856	.005∗	Support
H1d		PI	-.083	.040∗	.771	.002∗	.688	.007∗	Not support
H2a	SVQA	IM	.356	.003∗	/	/	.356	.003∗	Support
H2b		SA	.298	.001∗	.119	.004∗	.417	.002∗	Support
H2c		PI	.052	.073	.338	.004∗	.390	.001∗	Not support
H3a	IM	SA	.334	.003∗	/	/	.334	.003∗	Support
H3b		PI	.494	.001∗	.130	.002∗	.624	.002∗	Support
H4	SA	PI	.389	.001∗	/	/	.389	.001∗	Support

∗ The level of significance is 0.05.

The moderation effect test results showed that product involvement played a significant moderating role in the influence path of interactive narrative on immersion, SVQA on immersion, SVQA on satisfaction, SVQA on purchase intention, immersion on satisfaction, and immersion on purchase intention. Considering that SVQA has significant influence on purchase intention in the case of low-level product involvement, the practical impact of this path is limited due to the path coefficient is less than 0.1. Therefore, five verified moderation effect paths were finally determined. When the degree of product involvement level is high, SVQA has a stronger effect on immersion, SVQA has a stronger effect on satisfaction and immersion has a stronger effect on purchase intention. When product involvement level is low, interactive narrative has a stronger effect on immersion and immersion has a stronger effect on satisfaction. The moderation effect test of each influencing path is shown in Table 12.

**Table 12:** Results of moderation effect.

IV	DV	Low product involvement		High product involvement		Nested Model Comparisons	
		β	Sig.	β	Sig.	CMIN	Sig.
IN	SVQA	.724	.043∗	.760	.022∗	1.118	.290
IN	IM	.624	.001∗	.479	.001∗	8.417	.004∗
IN	SA	.413	.000∗	.319	.000∗	3.272	.070
IN	PI	-.106	.110	-.084	.081	.051	.698
SVQA	IM	.285	.003∗	.437	.004∗	7.983	.005∗
SVQA	SA	.233	.001∗	.368	.002∗	7.384	.007∗
SVQA	PI	.099	.023∗	-.045	.330	6.702	.010∗
IM	SA	.419	.003∗	.249	.003∗	10.372	.001∗
IM	PI	.358	.001∗	.596	.003∗	8.841	.001∗
SA	PI	.492	.002∗	.391	.001∗	1.494	.222

∗ The level of significance is 0.05.

## 5 Discussion

Mobile interactive video advertising perception model (MIVAPM) was proposed through qualitative exploration and quantitative verification by this research. It is found that as the variables, interactive narrative and SVQA effectively affect consumer behavior. Immersion and satisfaction play a good role as a mediator variable. Product involvement moderates half of the paths. According to the results of the model, the following three suggestions are proposed.

First, marketers should investigate the preferences of their target consumers and choose interactive narratives based on the findings. The results of this study show that the interactive narrative of mobile video interactive advertisements can directly affect the SVQA, immersion and satisfaction of customers. This result confirms that the practice of improving QoE by creating a better interactive narrative experience mentioned in previous studies is feasible (Amato et al., 2019). It also echoes what de Bruin et al. (2022) pointed out in their research on news media that the types of narrative and interactive functions will affect the degree of immersion that audiences can achieve. In addition, C. Gu et al. (2022) found that the effect of interactive narrative design would affect user satisfaction when studying the use of augmented reality technology to tell course stories. This study also proves that this relationship exists in mobile interactive movie advertisements. It is worth noting that although interactive narrative does not have a positive direct effect on purchase intention, there is a significant indirect effect. This result echoes what Teo et al. (2018) pointed out in their research on the use of augmented reality technology to promote cosmetics that interactive technology can be used to optimize shopping experience and increase consumers' purchase intention. Therefore, for designers, interactive narrative is very important. This study is the first one that focuses on mobile interactive movie advertising and proves that it is closely related to advertising design effect and consumer behavior. A good interactive narrative can effectively improve consumers' evaluation of the advertisement itself, and influence the subsequent purchase intention. Considering that the actual operation items of interactive narrative include “interactive node structure” and “user participation mode” (Ryan, 2006), designers need to incorporate the preferences of their target consumers to consider what story lines to frame the text and what interactive ways to express a story.

Secondly, improving SVQA can effectively help mobile interactive video advertisements achieve their marketing goals. Although SVQA cannot directly affect purchase intention, it can indirectly affect purchase intention through the complete mediation of immersion and satisfaction. The finding echoes previous studies that have shown that high-quality images increase consumers' purchase intention of sneakers (Teo et al., 2018). SVQA is the audience's judgment of video image quality, and the image quality can be directly improved through adjusting the objective parameters (Bampis et al., 2018). However, the cost of production and transmission determines the limit of video parameters. The cost of equipment, processing, and traffic usage would increase exponentially to achieve better image quality. Videos with better image quality are more likely to hog traffic bandwidth, resulting in stalling interaction and playback. Obviously, audience's dissatisfaction with slowly-buffered videos affects the user experience (Elkins, 2018). This study innovatively proposes a new solution to improve the audience's SVQA for mobile interactive video advertisements, that is to make it difficult for the audience to notice the possible defects in video quality through the design effect of high-level interactive narrative. In order to achieve sales objectives in retailing, the quality parameters of video should be improved within a reasonable range, and more attention should be paid to enhance the interactive narrative experience of advertising. The combination of these two methods is a cost-effective strategy.

Thirdly, based on the theoretical model established in this study, higher product involvement is not always better. This finding also corroborates Zhou and Xue (2019) research results on the matching of advertising types and different product involvement degrees. Therefore, designers can formulate design strategies for mobile interactive video advertisements based on current consumer involvement in the product. If the level of involvement is high, audience's SVQA of advertisements should be improved. As mentioned in previous studies, the degree of product involvement plays an important moderating role in the influence path involving purchase intention (Reyes-Menendez et al., 2020). This study fully confirms the role of this moderation effect in mobile interactive video advertising. Consumers' SVQA on immersion, SVQA on satisfaction, and the influence of immersion on purchase intention are strengthened in the context of high product involvement. This may be that the audience knows and needs the product very well, and watching the advertisement is more motivated by the actual product demand, rather than being attracted by the content of the advertisement itself. Audiences need to obtain more information about products through high-quality pictures, and the way of improving picture quality can achieve marketing objectives more efficiently. It is worth noting that the research of Rhee and Lee (2021) shows that although interactive experience strengthens the online purchase intention of consumers with high involvement degree, it reduces their offline purchase intention. This suggests that consumers may want to place an order immediately during watching interactive video advertisements on their phones, so the related function could be added in the advertisement design process. In addition, online interaction in social media can help consumers increase product involvement and purchase intention (Bazi et al., 2022). In order to further increase product involvement, advertisements are suggested to introduce more relevance between the product and the audience, or integrate marketing communication by introducing social interaction features of the media.

On the other hand, if the product involvement level is low, the designer can focus more on how to improve the audience's evaluation of the interactive narrative. In this way, the effects of interactive narrative on immersion and immersion on satisfaction are strengthened. In this case, the audience may be unfamiliar with the product or lack of experience, while their interest in advertising stems entirely from the production and design effects of advertising. This research result also echoes Y. K. Choi (2019) conclusion that consumers are more likely to be persuaded by the characters in advertisements when the degree of product involvement is low. It is worth noting that the approach of promoting advertising SVQA lowers the efficiency in promoting marketing. Geng and Chang (2022) points out that for consumers with low product involvement, perceived quality has a greater positive impact on consumer behavior than consumers with high product involvement. This indicates that consumers' perceived quality of advertising itself and perceived quality of products may have opposite trends under the moderation of product involvement. In addition, when the design strategy is adopted to match low product involvement, in order to maintain the efficiency of interactive narrative, the description of the relationship between the product and the audience should be reduced in the advertising content. Video advertisements should deliberately create a distance between the product and the audience, or emphasize that the product has a sense of alienation and mystery. In this way, the audience can put more energy into interactive narrative, and the design and content of advertising can attract the audience more easily, and make them gradually develop interest in the product and purchase desire.

## 6 Conclusion

### 6.1 Theoretical contribution

Using qualitative coding method, this study proposes ten main dimensions that need to be paid attention to in the design of mobile interactive video advertisements for marketing purposes. These dimensions integrate and expand the theoretical basis of interactive video media in the field of marketing, and lay a foundation for the research of interactive video advertising marketing. In previous studies, for example, Henehan et al. (2020) believed that consumers should be involved in advertisements and find the parts of their desirability of content in advertisements. J. E. Lee and Youn (2020) pointed out that brands and the quality of videos should be paid attention to in advertisements. These scattered proposed dimensions were explored without preconditions and included in the list of dimensions listed in this study. The results of this study are helpful for researchers to further establish theoretical models in specific situations.

In addition, the MIVAPM model proposed in this study effectively verifies the relationship paths between some dimensions in the list. The results confirm the value of interactive Narrative and SVQA as independent variables in marketing research. The results also prove the important mediating role of immersion and satisfaction, as well as the regulating role of product involvement. The theoretical results are the first to investigate audience's perception and preference of mobile interactive video advertising using a more systematic control variable method, which is helpful to promote the theoretical development of interactive video in the fields of digital marketing, e-commerce and social marketing.

### 6.2 Practical contribution

The audience's QoE of advertising is often one of the key points marketers want to improve, and therefore increase cost-performance and try new technical approaches (Sheeba and Maheswari, 2022). This study reaffirmed the importance of subjective video quality assessment for marketing in mobile interactive video advertising, and proposed a design method to improve interactive narrative and enhance objective quality parameters to jointly affect the QoE of advertising, which has the advantage of cost saving in practical management. This study proposes two advertising design routes that can be differentiated by the current level of product involvement. For products with high involvement level, improving objective quality parameters is more conducive to influencing consumers' purchase intention. On the contrary, low-involvement products should be more efficient and cost-effective by considering the design effect of interactive narrative. Designers can choose a more appropriate and cost-effective strategy based on the current involvement level of the product. The results of this study provide practical suggestions for the development of marketing in the direction of interactivity and digitalization. Especially in the context of the COVID-19 pandemic, traditional offline retail activities have been severely affected (Roggeveen and Sethuraman, 2020). There is a close relationship between the experience of online advertising and consumer behavior. The results of this study can help designers to grasp the design direction and elements. Mobile interactive video advertising is an online marketing method that integrates interactive and digital media features. Making more effective design strategies will help promote the diversified and sustainable development of marketing activities.

### 6.3 Research limitations and future studies

The research limitations of this study are as follows. The number of participants in the questionnaire ranged from 15 to 29 years old, and the number of participants in each age group was roughly the same but not completely evenly distributed. The subjects were recruited from northern Taiwan through the public community platform method, and no other regional groups were involved. In future research, mobile interactive videos for specific purposes can be tested. For example, as a new form of media applied in learning, cognition and other fields compared to traditional media effect difference. On the other hand, the effect of gender or age on mobile interactive video advertising can be further tested. In addition, due to the limited number of samples produced, the complexity of the model framework and the physical effort required by respondents to participate in the survey, this study only conducts selective quantitative verification of the hypothetical model obtained from qualitative analysis. Future research can further test the correctness of this theoretical model through quantitative research.

## Declarations

### Author contribution statement

Chao Gu: Conceived and designed the experiments; Performed the experiments; Analyzed and interpreted the data; Wrote the paper.

Shuyuan Lin: Conceived and designed the experiments; Analyzed and interpreted the data.

Jie Sun; Wei Miao: Analyzed and interpreted the data; Wrote the paper.

Chun Yang; Jiangjie Chen; Qianling Jiang: Performed the experiments; Contributed reagents, materials, analysis tools or data.

Wei Wei: Performed the experiments; Contributed reagents, materials, analysis tools or data.

### Funding statement

This work was supported by Changshu Institute of Technology [grant number KYZ2020065Q].

### Data availability statement

Data will be made available on request.

### Declaration of interest's statement

The authors declare no conflict of interest.

### Additional information

No additional information is available for this paper.

## Figures and Tables

**Table:** 

Constructs	Coding	Items
Interactive narrative	IN1	I think the interaction of the AD is meaningful.
	IN2	I think this AD and I have a high degree of interaction.
	IN3	I think the AD gave me a new perspective.
	IN4	I think the plot and interaction of the AD are reasonable.
	IN5	I think the interactive experience provided by this AD is diverse.
	IN6	I think the AD is interactive.
	IN7	I think the manipulation of the AD is reasonable.
Subjective video quality assessment	SVQA1	I think the imaging quality of this AD is good.
	SVQA2	I think the design of this advertisement is exquisite.
	SVQA3	I think the advertisement is aesthetically pleasing.
	SVQA4	I think this advertisement is suitable for mobile phone.
	SVQA5	I think the colors in this AD are nice.
	SVQA6	I think the rhythm of the video is smooth.
Immersion	IM1	I was not aware that I was using any device to control it.
	IM2	I think this AD is very immersive for me.
	IM3	I'll be interested to see how this advertising thing goes.
	IM4	I'd love to see this AD on the full screen.
	IM5	I'm really focused on watching this AD.
	IM6	I think watching this AD is experiencing something rather than just doing something.
	IM7	I think the AD has a puzzle element.
Satisfaction	SA1	I think the plot of the AD is smooth.
	SA2	I think this AD has an appropriate advertising length.
	SA3	I think the advertisement is creative.
	SA4	I think this AD gives a good user experience.
	SA5	I think the AD provides an appropriate guide to how the interactive process works.
	SA6	I would like to share this AD.
Purchase intention	PI1	If I have a need, I'm more likely to buy the advertised product.
	PI2	I am willing to keep buying the advertised products.
	PI3	I will buy the product in the advertisement.
	PI4	I will probably buy the advertised product.
	PI5	If the price is the same, I will choose the advertised product.
	PI6	I'd like to recommend the advertised product to my friend.
